# Introduction of the targeted alpha therapy (with Radium-223) into clinical practice in Japan: learnings and implementation

**DOI:** 10.1007/s12149-018-1317-1

**Published:** 2018-11-27

**Authors:** Makoto Hosono, Hideharu Ikebuchi, Yoshihide Nakamura, Sachiko Yanagida, Seigo Kinuya

**Affiliations:** 1 Institute of Advanced Clinical Medicine, Department of Radiology, Kindai University Faculty of Medicine, 377-2 Ohno-Higashi, Osaka-Sayama, 589-8511 Osaka Prefecture Japan; 2 Japanese Society of Nuclear Medicine, 2-28-45 Honkomagome, Bunkyo-ku, Tokyo, 113-0021 Japan; 3 Japan Radioisotope Association, 2-28-45 Honkomagome, Bunkyo-ku, Tokyo, 113-8941 Japan; 4 Department of Nuclear Medicine, Faculty of Medicine, Medical, Pharmaceutical and Health Sciences, Kanazawa University, 13-1 Takaramachi, Kanazawa, 920-8641 Ishikawa Prefecture Japan

**Keywords:** Ra-223, Targeted alpha therapy, Alpha-emitters, Radionuclides, Metastatic castration-resistant prostate cancer

## Abstract

Radium-223 dichloride (Ra-223) is the first targeted alpha therapy approved for the treatment of patients with castration-resistant prostate cancer (CRPC) with bone metastasis. Ra-223 improved overall survival in the international Phase III ALSYMPCA (ALpharadin in SYMPtomatic Prostate Cancer) study. Ra-223 was also demonstrated to be efficacious and safe in Japanese patients in Phase I and Phase II clinical trials. Ra-223 was approved in Japan for the treatment of patients with CRPC with bone metastasis in 2016. The conduct of clinical studies with radionuclides in Japan involves mandatory compliance with local and international regulations pertaining to radiation protection. Without an existing Japanese framework for the handling of α-emitters in clinical practice, we encountered many challenges to initiate the clinical studies. Therefore, we started on a project to determine best practice on the use of Ra-223 in clinical studies. For this project, we evaluated all applicable laws and regulations on the use of radionuclides in medicine, then examined whether and how the α-emitter Ra-223 could meet these legal and regulatory requirements. This included how to approach the matter of discharging patients administered Ra-223 from hospital and radiation protection for caregivers, general public and medical care professionals. Subsequently, we published *Manual on the proper use of radium-223 dichloride injection in clinical trials* that summarized the essential requirements necessary to allow the safe use of Ra-223 in clinical trials in Japan. As the result, we succeeded in demonstrating that clinical trials of an α-emitter, Ra-223, could be implemented safely in Japan. Our experience in Japan highlights the importance of a multidisciplinary team-based approach and continued professional training in a clinical setting. This article summarizes the rationale behind the development of this manual. We hope that by sharing our experience and information, we can help other countries considering the introduction of radionuclides for clinical use, and support the future development of radionuclide therapies in a safe and effective manner.

## Introduction

Radium-223 dichloride (Ra-223) is a targeted α-emitting agent used to treat bone metastasis associated with prostate cancer. Ra-223 improved overall survival in the international ALSYMPCA (ALpharadin in SYMPtomatic Prostate Cancer) study [1]. Ra-223 was also demonstrated to be efficacious and safe in Japanese patients in Phase I and Phase II clinical trials [2, 3]. Ra-223 was approved in Japan for the treatment of patients with castration-resistant prostate cancer (CRPC) with bone metastasis in 2016.

Ra-223 was introduced to Japanese clinical practice 2–3 years after its introduction in Western countries [4], and use of Ra-223 has increased rapidly in Japan since its launch. Introduction of Ra-223 to Japan has also increased clinicians’ willingness to use α-emitters, which were conventionally considered too hazardous for use in clinical practice. This perception has changed since regulatory approval of Ra-223, and appropriate rules and regulations have now been developed for handling this nuclide.

Although therapy using radionuclides is shown to benefit patients, those who are treated with radionuclides may become a source of radiation exposure to other people. Therefore, to use radionuclides in clinical trials, there must be compliance with local and international regulations on radiation protection. Also, the exposure dose must be reduced, and exposure to other patients, family members, caregivers, and the general public must be prevented.

Although regulations are in place for the release of patients administered β-emitters from controlled areas in Japan (Notice No. 70 from the Safety Division, Pharmaceutical and Medical Safety Bureau, Ministry of Health, Labour and Welfare, dated June 30, 1998, Notice from the Head of the Safety Division, Pharmaceutical and Medical Safety Bureau hereafter referred to as “PMSB Notice No. 70”), to introduce α-emitters we needed to examine safety measures based on the basic principles of radiation protection. This included the need for hospitalization in a medical ward for radiotherapy, and regulations related to the release of patients from controlled areas.

There were many challenges associated with the introduction of Ra-223 in Japan. First, there was no framework for handling α-emitters in clinical practice to ensure compliance with the complicated laws and regulations in Japan. Moreover, due to concern over greater internal exposure from their higher linear energy transfer, α-emitters are subject to stricter management in terms of concentration limits than either β-emitting or γ-emitting nuclides.

We conducted a project titled *Community healthcare based development and research promotion project*, which was supported by The Health Labor Sciences Research Grant, to provide best practice on the use of Ra-223. This project involved an evaluation of all applicable laws and regulations in Japan relating to the introduction of Ra-223. We subsequently published an instruction manual summarizing the main requirements necessary to ensure radiation protection in clinical practice. Compliance with these requirements was instrumental to starting the conduct of clinical trials of Ra-223 in Japan.

This article summarizes the rationale behind the development of *Manual on the proper use of radium-223 dichloride injection in clinical trials* [5]and its later version,*Manual on the proper use of radium dichloride (Ra-223) injection (Safety management)* [6]. Both these manuals are based on our extensive discussions of Ra-223 radiation protection and management. This included assuming a dispersion constant for Ra-223 based on the properties of the immediate Rn-219 progeny, and using the observed dose rate of an Ra-223 vial when estimating external radiation. By sharing this experience and information, we hope to inform those considering the introduction of Ra-223 and other radionuclides into other countries in the future. Definitions for terms used in this article are summarized in the “appendix”. Items in the manual related to the safe handling of radiation originate from the fundamental requirements for radioprotection as recommended by the International Commission on Radiological Protection (ICRP), and are also derived from the International Basic Safety Standards of the International Atomic Energy Agency (IAEA) [7].

## Characteristics of Ra-223

**Fig. 1:**
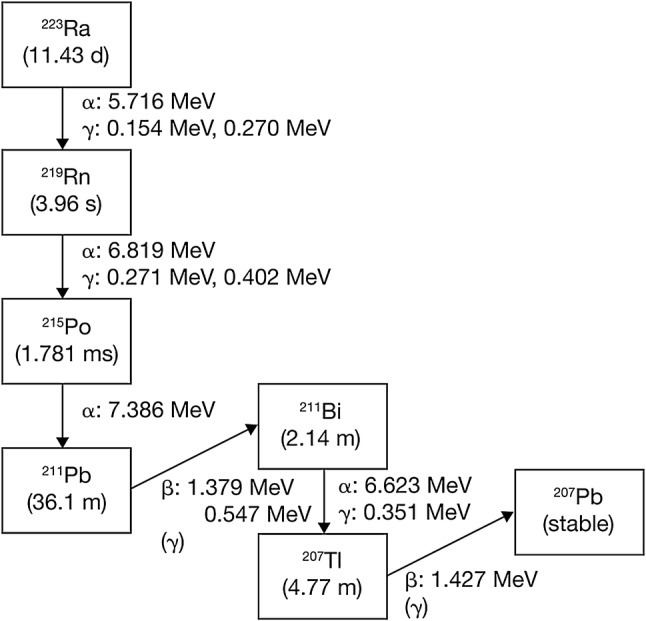
Radioactive decay of Ra-223

Figure 1 shows the principal decay series for Ra-223. The progeny of Ra-223 have a relatively short physical half-life. Lead-207 (Pb-207), a stable nuclide, is generated via four alpha and two beta decays. Alpha and beta radiation generated in the decay series of Ra-223 also generates gamma radiation.

In the first step, Ra-223 undergoes alpha decay and generates radon-219 (Rn-219), a colorless and odorless gas at room temperature. Radon can cause internal exposure when it disperses into the air, and was a concern for handling of Ra-223. According to its International Chemical Safety Card (ICSC No. 1322), Rn-222, a nuclide that shares gaseous properties with Rn-219, has a boiling point of − 62 °C, a melting point of − 71 °C, and a density of 9.73 g/L. Radon’s solubility in water is 22.68 mL in 100 mL at 25 °C (0.2268 mL/mL), which is substantially higher than that of two other noble gases, xenon and krypton. The Rn-219 content per 1 MBq of Ra-223 injection is equivalent to a maximum of 2.13 × 10^− 13^ mL under standard conditions. Considering the solubility of radon in water, which is 0.2268 mL/mL, the maximum content 2.13 × 10^− 13^/mL is one/1.06 × 10^12^ of the solubility when it is in a solution, which is extremely low.

The noble gas element, Rn-219, has an extremely short half-life (3.96 s) and decays into non-dispersive polonium-215 (Po-215). Under normal conditions, it is extremely unlikely that Rn-219 would disperse into the atmosphere.

In vivo, based on the data from γ-camera images, administered Ra-223 rapidly distributes into bone, especially where bone remodeling is activated, including areas affected by bone metastasis. Ra-223 distributed in bone decays into Rn-219 with a half-life of 11.43 days. As mentioned above, Rn-219 decays into Po-215 with a short half-life of 3.96 s. Polonium, which has a biological half-life of 50 days, has a strong affinity for tissues of the liver, kidney, and spleen. The high affinity of lead for bone tissue is also well-established and preclinical studies show that not more than 2% of Ra-223 progeny is released from bone [8]. Based on these data, it is likely that most Ra-223 progeny distributed in the body remain in the bones.

As mentioned above, even considering the effect of Rn-219 as one of these progeny, when handling Ra-223, a dispersion rate of 0.001 (which is applied to radionuclides in the Medical Care Act) would be a conservative assessment.

## Facility management

### Laws and regulations in Japan

In Japan, the conduct of clinical trials, including those using radionuclides, is subject to the stipulations of the Medical Care Act. Moreover, other laws and ordinances relating to the prevention of radiation hazards in participating healthcare providers also apply, e.g., for national institutions, the National Personnel Authority Rule No. 10 − 5 [9] (National Public Service Act) details the prevention of radiation hazards for staff members; meanwhile, for the general public, and private and civil institutions the stipulations of the Ordinance on Prevention of Ionizing Radiation Hazards [10] (Industrial Safety and Health Act) apply. The use of radionuclides for non-medical purposes, or outside of clinical trials, is also subject to the Act on Prevention of Radiation Hazards due to Radioisotopes, etc.[11].

### Facility requirements

As stipulated in Article 15 of the Medical Care Act and Articles 24 and 28 of the Ordinance for Enforcement of the Medical Care Act, when Ra-223 is used in hospitals, written notification must be submitted to the governors of prefectures within which the hospitals are located.

### Points of notification

As stipulated in Medical Care Act, all facilities must provide information regarding the estimated maximum quantity of radionuclides used in 1 day, 3 months, and 1 year, as well as the estimated maximum quantity in storage based on effective dose, leakage radiation dose, and hypothetical concentrations in drain water, room air, and discharged air to local government. In addition, there are stipulated compliance requirements for administrators of hospitals or medical facilities on the handling of medical radionuclides (see Table 1).

**Table 1:** Criteria for facilities using radioactive substances

	Medical Care Act
Facilities required	Rooms especially designated for the use of medical radionuclides
	Storage facilities
	Waste management facilities
Dose limits for controlled areas	Effective dose: not exceeding 1.3 mSv per 3 months Surface contamination density: not exceeding 0.4 Bq/cm^2^ (nuclides that emit alpha particles) Concentration in the air: average concentration over 3 months not exceeding 1/10 of concentration limit
Places in facilities where people are constantly entering	Effective dose at outside walls, etc, not exceeding 1 mSv per week
Business boundaries (including areas in the hospital that might be accessed by treated patients)	Not exceeding 250 µSv per 3 months
Exposure of inpatients	Not exceeding 1.3 mSv per 3 months

Modified from Ordinance for Enforcement of the Medical Care Act, Article 30 [22]

### Restrictions on the hospital admission of patients

The following restrictions on the hospital admission of patients are stipulated in PMSB Notice No. 70: (1) patients receiving medical treatment with radionuclides must not be admitted to any wards other than isolation rooms (rooms for patients administered radiopharmaceuticals); (2) patients not receiving medical treatment with radionuclides must not be admitted to isolation rooms. In addition, medical radionuclides must be used in rooms especially designated for this purpose.

The Ministry of Health, Labor, and Welfare stipulates minimum release criteria that are required for patients receiving radionuclide therapies, including for iodine, strontium, and yttrium. As these release criteria were established on the basis of clinical trial results, preliminary release criteria for Ra-223 were needed to start conducting clinical trials of this agent. These preliminary release criteria were obtained based on data from various simulations that are described later in this article.

### Safety management of Ra-223 injections

When using Ra-223, all standards established in related laws and ordinances must be observed and effective safety management must be ensured. Ra-223 must be handled and stored in an appropriate manner and its location clearly recorded.

## Record-keeping

The following are required items in usage records as stipulated in PMSB Notice No. 70: (1) product specifications, (2) date of arrival, (3) date of use, (4) quantity used, (5) quantity remaining, (6) user, (7) name of patient, (8) date of storage or waste management of medical waste, and (9) radiation levels during storage or waste management of medical waste. Storage records are kept to confirm that the quantity stored in designated facilities does not exceed the maximum permitted amount.

### Measurement at locations with a high risk of radiation hazards and strage of records

Radiation exposure and radioactive surface contamination levels must be measured at certain locations related to the use of the radionuclide before starting treatment and no later than 1 month after starting treatment (6 months for some locations). These locations include rooms where the radionuclide is used, the outside walls of these rooms, storage and waste management facilities for medical waste (including drainage appliances), boundaries of controlled areas, areas that might be accessed by treated patients, isolation rooms, and site boundaries. These records must be kept for 5 years. Dose rate in the area is measured based on the 1 cm dose equivalent rate or the 70 µm dose equivalent rate in locations that may exceed the 1 cm dose equivalent rate tenfold. The dose rate and any contamination by radionuclides is measured using a measurement instrument.

### Measurement of exposure doses to medical staff involved in radiation therapy and retention of records

Effective and equivalent doses of radiation exposure (external and internal) to medical staff are given in the Ordinance for Enforcement of the Medical Care Act, Article 30. Using these results, doses are estimated as specified by the Minister of Health, Labor, and Welfare Ministry.

### Records related to the release of patients

The dose received by patients and dose rate from the patients at discharge must be recorded. Discharge date/time must also be recorded and records kept for 2 years.

## Restrictions on places of use

According to the Ordinance for Enforcement of the Medical Care Act, Article 30, medical radionuclides must be used in areas especially designated for this purpose or in isolation rooms. As a general rule, Ra-223 is for use in areas especially designated for the use of medical radionuclides. When using medical radionuclides, it is also necessary to provide storage and waste management facilities for medical waste. (See Table 1).

### Concentration limits and management of radioactivity in room air, discharged air and drain water

Facilities must provide information about the type and quantity of radionuclides used to the local government office in advance. Furthermore, they are also required to estimate the concentration in the air of controlled areas, discharged air, and drain water in controlled areas and to ensure these results are within the limits of the legal level (see Table 2). If more than one radionuclide is used, the sum of the ratios of the concentration limits (estimated value/concentration limit) should not exceed 1. If a facility wants to modify its radionuclide use, it is required to re-estimate concentrations in the room air, discharged air, and drain water. It must also notify the local government; this system is unique to Japan and demonstrates the importance of planning and effective management to ensure the safe handling of radioactive products.

**Table 2:** Concentration limits of Ra-223 and other medical radionuclides

	Concentration limit (Bq/cm^3^)		
Type of radionuclide	Room air	Drain water	Discharged air
Ra-223	4 × 10^− 6^	5 × 10^− 3^	2 × 10^− 8^
I-131	2 × 10^− 3^	4 × 10^− 2^	1 × 10^− 5^
Y-90	1 × 10^− 2^	3 × 10^− 1^	8 × 10^− 5^
Sr-89	1 × 10^2^	3 × 10^− 1^	1 × 10^− 4^
Tc-99m	1 × 10°	4 × 10^1^	9 × 10^− 3^

Modified from Ordinance for Enforcement of the Medical Care Act, Article 30 [22]

Contamination, ambient dose, and concentration in the air are monitored with reference to the following measurements: (1) surface contamination, (2) 1 cm dose equivalent rate, (3) concentration of radioactive substances in the air. Measurements are made at least once a month (once every 6 months in specified locations). The records of the results must be retained for 5 years. Precautions must be taken to protect against possible contamination in areas at high risk; these include covering such areas, in advance, with materials such as water-absorbent, polyethylene-coated filter paper.

The determination of radionuclide levels in room air, discharged air and drain water can be outsourced to a reliable external company that specializes in such assessments.

### Radiation protection

#### Radiation protection when using Ra-223

Clinical trials should be performed in compliance with the ALARA (As Low As Reasonably Achievable) principle. When handling Ra-223 in a clinical trial, three principles of protection from external exposure must be observed to reduce exposure. These are: to reduce working time as much as possible, to maintain an adequate distance from the source, and to use appropriate shielding. Dose rates measured when handling Ra-223 without shielding are summarized in Table 3.

**Table 3:** Dose rates measured from glass vials of Ra-223 without shielding

Distance from vial	Dose rate (µSv/h/MBq)
1 m	< 0.1
10 cm	< 5
Surface	< 100

Ra-223 is supplied for clinical trial use as a colorless and clear aqueous solution in 20 mL vials. Each vial contains 6.6 MBq of radioactivity, i.e., 1.1 MBq/mL (test day). Data in this table reflect the updated National Institute of Standards and Technology standards (2014) [23]

When preparing Ra-223 and disposing of waste after administration to patients, a laboratory coat, gloves, and other means of protection must be worn.

If the hands, skin, face, eyes, or any other parts of the body are accidentally contaminated by Ra-223, the affected parts should be cleaned immediately with water. Contamination by Ra-223 can be removed using a solution of 0.01 M ethylene-diamine-penta-acetic acid or other complexing agents.

After Ra-223 use, working sites in controlled areas should be assessed to ensure that there is no contamination. As Ra-223 emits α-, β-, and γ-radiation, detectors that can identify any of these should generally be used to detect surface contamination; however, using β-particle detectors instead of α-particle detectors offers the following advantages: a high counting efficiency for Ra-223 contamination and a low risk of contaminating the detector as the distance from the surface being assessed is greater than the range of alpha emissions in the air.

### Exposure of healthcare providers (external and internal exposure)

The dosage of Ra-223 varies between patients; however, based on a dosage of 11 MBq^1^, the external exposure dose for healthcare providers can be calculated according to time procedures and distance from radiation source, as shown in Table 4. As shown in Fig. 1, Ra-223 forms a decay series in which the parent nuclide and the progeny are in a state of radiation equilibrium (secular equilibrium) (see Figure S1 in the “appendix”). At equilibrium, the radioactivity of each progeny is equal to the radioactivity of the parent nuclide. For assessment of the exposure dose, a constant for each progeny was added to the effective dose rate constant of Ra-223 as published in Radioisotope Pocket Data Book (11th ed.), i.e., 0.0454 [µSv × m^2^ × MBq^− 1^× h^− 1^] was used as the value for Ra-223 equilibrium.

**Table 4:** External exposure dose for healthcare providers

	Effective dose (whole body) (per person)			Skin (per person)			Dose limit	
	Working time (min.)	Distance (cm)	Exposure dose (mSv)	Working time (min.)	Distance (cm)	Exposure dose (mSv)	Effective dose limit (whole body)	Equivalent dose limit
Preparation	10	50	3.03 × 10^− 4^	10	1	0.757	Men 50 mSv/year 100 mSv/5 years Women 5 mSv/3 months	500 mSv/year
Administration	5	50	1.51 × 10^− 4^	5	1	0.378		

As the maximum dosage for clinical trials in Japan is 110 kBq/kg per application, the total dosage is assumed to be safe if the patient’s body weight does not exceed 100 kg

Based on the Ministry of Health and Welfare Notification No. 398 of December 26, 2000 [12], internal exposure (the effective dose, *E*, measured in mSv) is calculated according to the formula below [13]:

$$E=e \times I$$

Where *I* is the amount of medical radionuclide used (Bq) taken in by inhalation, and:

$$I={\text{1}}.{\text{2}} \times {\text{1}}{0^{\text{6}}} \times C \times t$$

1.2 × 10^6^ = Intake rate of air inhaled by an adult in 1 h (cm^3^/h)

*C* = Radioactive concentration in the air (Bq/cm^3^)

*t* = Working time (h)/week

*C* = *A* × dispersal rate × days of use in 1 week/ (*V* × 10^6^ × 8 [h] × days of use in 1 week)

*A* = Estimated maximum use in 1 day (Bq)

*V* = Discharged air indoors (m^3^/h), 8 operating hours/day

For Ra-223, the following values apply:

*A*: 11 MBq, dispersal rate: 0.001, discharged air indoors: 1 day, *V*: 560 (m^3^/h), days of use during 1 week: 5 days, working time: 10 min (0.167 h), *℮* (effective dose coefficient if Ra-223 was taken in by inhalation): 5.7 × 10^− 3^ (mSv/Bq). Thus, internal exposure (effective dose) for Ra-223 is as follows:

*C* = 11 × 10^6^ × 0.001 × 5 / (560 × 10^6^ × 8 × 5) = 2.46 × 10^− 6^ (Bq/cm^3^)

*I* = 1.2 × 10^6^ × *C* × 0.167 × 5 = 2.46 (Bq)

*E* = *e* × *I* = 5.7 × 10^− 3^ × 2.46 = 1.40 × 10^− 2^ (mSv)

#### Education and training

To ensure the safe handling of radiation, clinical trials should be carried out by physicians with sufficient knowledge of radiation best practice, as well as other personnel who have received adequate training. Clinical trials should be conducted in facilities which can ensure radiation safety.

#### Important points after administration

### Discharge of patients from hospital

Advice concerning the release of patients who have been administered unsealed radionuclides is covered in publications by the ICRP [14]. The following points should be considered when evaluating radiation exposure doses from patients who were administered radioactive substances:

#### General public dose limit: 1 mSv/year

The recommended general public dose limit (1 mSv/year) is taken from the ICRP Publication 60 (1990) [15]. Under special circumstances, higher values are permitted each year, provided that the average over 5 years does not exceed 1 mSv per year. Although not yet incorporated into national laws and ordinances, the ICRP Publication 103 (2007) [16] will be the successor to Publication 60, but the general public dose limit of 1 mSv/year will not change.

#### Cumulative dose value for caregivers: 5 mSv

With regards to the exposure of caregivers, volunteers, etc., Clause 95 of the ICRP Publication 73 (1996) [17] classifies the exposure of friends and relatives who assist in nursing and caring for patients as “medical exposure” and suggests that “dose constraints in the order of several mSv per case are reasonable”. On the other hand, the IAEA’s International Basic Safety Standards (1996) [7] state that the dose limits established in this section are not applicable to caregivers, i.e., individuals knowingly exposed to radiation while voluntarily (not on an employment or work-related basis) helping with nursing, attending to, and caring for patients who receive medical check-ups or therapies, or to visitors of these patients. Nevertheless, the dose to which any caregiver or visitor is exposed must be limited so as not to exceed 5 mSv per activity during the patient’s check-ups or therapies. The dose to which children may be exposed, when visiting patients who have been administered radioactive substances, must be less than 1 mSv.

### Exposure factor

The duration of contact and the distance from the patient during this time influence the exposure dose. Consequently, the exposure factor,^**^ which needs to be taken into account when assessing the exposure dose of third parties, must be set by considering each individual’s degree of involvement.

#### Exposure factor for caregivers: 0.5

Based on measured values for patients administered radiopharmaceuticals, applying an exposure factor of 0.5 is reported to be reasonable in cases where careful nursing is required [18]. The results of surveys conducted in Japan to determine the exposure dose from treated patients also suggest that a factor of 0.5 is appropriate [19]. Thus, the exposure factor of 0.5 is used when assessing the dose for caregivers after patients are released or discharged from the facilities.

#### Exposure factor for the general public: 0.25

Based on measured exposure doses of patients’ families in typical households, an exposure factor of 0.25 is reported to be appropriate [18]. Accordingly, an exposure factor of 0.25 is used when assessing the dose for (non-caregiving) family members and the general public, after the patients are released and discharged.

#### Calculation of external exposure dose

The external exposure dose is calculated using the following formula [13]:

$$I=A \times C \times {F_{\text{a}}} \times t/L$$

where

*I*: Effective dose at calculation point (µSv)

*A*: Radioactivity (MBq)

*C*: Effective dose rate constant of radiation source (µSv × m^2^ × MBq^− 1^ × h^− 1^)

*F*_a_: Effective dose transmission rate (in cases of multiple shielding, the sum of the transmission of each shield is used as the total transmission rate)

*t*: Duration of use (h)

*L*: Distance from radiation source to calculation point (m)

To calculate an exposure dose for third parties after a patient treated with Ra-223 has been released from hospital and discharged to go home, the dose rate and cumulative dose at a distance of 1 m from the patient’s body surface are used.

#### Amount of residual radioactivity inside the body

The amount of radioactivity inside the body of patients administered radiopharmaceuticals decreases according to the physical half-life of the nuclide and its metabolism and excretion by the patient (biological half-life). Therefore, the assessments are conducted based on the effective half-lives, where both the physical and biological half-lives are taken into account. However, there are large inter-individual variations in the biological half-life of radioactive substances and these can be affected by disease states etc. For this reason, only physical half-life is used so a safe estimate is obtained for the amount of residual radioactivity inside the body.

#### Assessment of internal exposure

In general, radioactive substances administered to patients are excreted from the body via respiration, urine, feces, sweat, saliva, breast milk, etc. It is therefore possible that these substances constitute a source of internal exposure for families and the general public. Internal exposure can be limited by taking appropriate hygiene measures and, in breast-feeding mothers, by abstaining from breast feeding for a certain period of time.

*******Assessment of doses that caregivers and the general public receive from patients administered Ra-223(heading

#### Assessment of external exposure doses

To assess the external exposure by Ra-223 inside the patient’s body, a decrease by physical half-life was assumed, without considering the excretion of Ra-223.

Cumulative dose of external exposure per 1 MBq amount of residual radioactivity inside the body.

1 [MBq] × 0.0454 [µSv × m^2^ × MBq^− 1^ × h^− 1^] × (11.43 [d] / 0.693) × 24 [h/d] = 18.0 [µSv]

Dose rate at a distance of 1 m from a patient administered 11 MBq*

11 [MBq] × 0.0454 = 0.499 [µSv/h]

Caregiver’s annual cumulative dose (6 injections 11 MBq,^2^ assuming an exposure factor of 0.5).

18.0 [µSv/MBq] × 11 [MBq/application] × 6 [injection(s)/year] ÷ 1000 [µSv/mSv] × 0.5 = 0.59 [mSv/year].

General public annual cumulative dose (6 injections 11 MBq, assuming an exposure factor of 0.25).

18.0 [µSv/MBq] × 11 [MBq/application] × 6[injection(s)/year] ÷ 1000 [µSv/mSv] × 0.25 = 0.30 [mSv/year].

From the above, the exposure for caregivers and the general public resulting from the six 11 MBq doses is below the recommended limits (5 mSv/year and 1 mSv/year, respectively); thus, release from hospital and return home can be approved, provided that written and oral advice, and instructions related to daily life are given. If the dose planned in the clinical trial is more than six doses of 11 MBq/day or the daily dosage is above 11 MBq, it is necessary to reconfirm that there is no deviation from the above principles regarding dosage and administration.

#### Important points for patients and family members

Because some radioactivity exists in bodily fluids (mainly in the blood), urine and feces after administration of Ra-223, it is important that the following points are communicated in writing and understood by both the patient and family members. These points should be performed during the first week after each administration of Ra-223:

#### General advice

In case of bleeding, wipe off the blood completely, using toilet paper, and flush down the toilet.

If there is a risk that the patient’s clothes, etc, have been contaminated with urine/feces, wear disposable rubber gloves when handling these items.

If your hands or skin come into contact with the patient’s blood, wash the areas thoroughly using soap.

#### *****Patients and caregivers are to refrain from sexual activities(not heading).

There is no special limitation on contact between the treated patient and children or pregnant women.

If possible, the patient should take a bath or shower at the end of each day. The bath tub/shower cubicle should be washed and scrubbed thoroughly afterwards.

#### Advice for handling clothes

Wash the clothes worn by the patient separately from those of other family members.

Any clothes and bedclothes contaminated with blood or urine should be laundered and rinsed thoroughly.

******Advice relating to urination, defecation, and vomiting(heading)

Men should urinate in a sitting position.

Flush the toilet twice after use.

For urine or feces spillages, wipe thoroughly, using toilet paper, and flush down the toilet.

Wash hands thoroughly after using the toilet.

Wash hands and skin thoroughly using soap and rinse thoroughly with water after coming into contact with a patient’s urine, feces, vomit, etc.

#### Important points for medical care professionals

It is essential for healthcare providers participating in Ra-223 clinical trials to understand the principles of radiation safety and the pharmacokinetic properties of Ra-223. They must also be able to explain the principles of radioprotection (see above) to patients and family members and to ensure effective safety management practices in medical institutions. It is also important that physicians with expertise in conducting Ra-223 clinical trials provide appropriate training of other healthcare providers and develop a cooperation framework within medical institutions.

In the event of a medical emergency, appropriate medical measures should take priority over complying with the matters related to radioprotection described above.

#### Disposal of radioactive products

As stipulated in the Medical Care Act, materials contaminated with Ra-223 are categorized as radioactive medical waste, and the waste should be stored in the appropriate storage facilities at medical institutions.

Prior to its marketing approval, materials contaminated with Ra-223 were not yet considered medical radioactive waste and hospitals were required to store these contaminated objects at their own facilities until approval was granted. Now that Ra-223 has been approved, materials contaminated with Ra-223 are considered radioactive medical waste and collected by Japan Radioisotope Association.

For handling of diapers, urine collection packs, and other objects contaminated with human urine, feces, and blood in facilities, refer to *Handling of diapers etc. of patients administered radiopharmaceuticals* [20]. When the level of radioactivity is confirmed to be equal to background radiation, the radioactive medical waste can be removed from the facility.

## Conclusions and future perspectives

Recently, the use of radionuclides has become more prevalent, with the development of agents labeled with iodine-131, yttrium-90, and lutetium-177 [21]. In particular, there has been considerable interest in α-emitters [21], such as Ra-223, bismuth-213, and actinium-225 [22].

The laws and regulations regarding the use of radiotherapy in Japan are generally comparable with those overseas. However, the introduction of Ra-223 in Japan was challenging, as the Japanese legislation system was thought too strict to allow use of alpha-emitters in clinical medicine. One example was that the concentration limits of unsealed α-emitters were too strict to be used for medical treatment. We have addressed these challenges using a stepwise scientific approach to establish standards and rules that allow Ra-223 to be used effectively and reliably in clinical practice. As a result, we demonstrated that clinical trials with α-emitters could be implemented safely by appropriate handling and management of radionuclides in accordance with the principles of radiological protection.

Initially, we drafted *Manual on the proper use of radium-223 dichloride injection in clinical trials*. Subsequently, we conducted studies to establish criteria associated with the release of patients administered Ra-223 [23]. These criteria were subsequently issued to medical facilities as a notice by the Ministry of Health, Labor, and Welfare. Information acquired after issuing these criteria were then integrated into the initial draft to produce the updated *Manual on the proper use of radium dichloride (Ra-223) injection* with added content on clinical use. *Manual on the proper use of radium dichloride (Ra-223) injection* was developed by related societies and provides significant help for the establishment of safety measures in medical facilities [4, 5, 7, 23, 24]. This manual was created to demonstrate how to ensure radiation safety and address any challenges associated with use of Ra-223 in clinical practice, but does not stipulate strict regulations and rules.

To further develop internal radiation therapy, it will be necessary to prepare and observe thorough and appropriate management principles for safe handling. To ensure appropriate handling, each member of a multidisciplinary team must have clear responsibilities, healthcare professionals must collaborate with one another, and systematic and continuous training of team members must be routine.

In summary, a multi-disciplinary team and continued professional training are key to successfully using Ra-223. We hope that our experience provides useful information to other countries on the safe handling of α-emitters in the medical field.

## Appendix

### Definitions used in Japanese regulations

#### Dose rate

Quantity of radiation absorbed per unit of time.

#### Effective dose

The cumulative dose for each tissue and organ in the human body after correction using the respective tissue weighting factor, thus indicating the size of the effect when the whole body is exposed uniformly. The effective dose, *E* is expressed by the following formula:

$$E=\sum\limits_{T} {{w_T}\sum\limits_{R} {{w_R}{D_{T \cdot R}}} }$$ or $$E=\sum\limits_{T} {{w_T}{H_T}}$$

Where *H*_T_ is the equivalent dose in tissues or organs (*T*) and *W*_T_ is the tissue weighting factor. The unit of effective dose is usually the sievert (Sv).

#### Effective dose rate constant

Constant for determining the effective dose per hour at a distance of 1 m with an unshielded point source of radioactivity of 1 MBq (µSv × m^2^ × MBq^− 1^ × h^− 1^).

#### Dose equivalent

Specifies, for the purpose of radioprotection, a quantity for assessing the effect of exposure using a common measure of radiation type and form of exposure. The unit of dose equivalence is the sievert (Sv).

#### 1 cm dose equivalent (rate)

As the effective dose cannot be measured directly, the International Commission on Radiation Units and Measurement (ICRU) proposed two quantities relating to practical measurements of external exposure, i.e., the ambient dose equivalent *H**(d) for work environment monitoring, which always corresponds to higher values than effective doses under ordinary exposure conditions and therefore provides a conservative estimate and secondly the personal dose equivalent *H*_*p*_(d), used for monitoring personnel. Both are used internationally, including in Japan. If tissues in the human body are exposed to X-rays or γ-rays, the greatest impact of the exposure dose is on tissues at a certain depth, rather than on the body surface. If exposure doses at a depth of 1 cm from the body surface are used as assessment standards, they will always be greater than effective doses, permitting exposure management with a safety margin. The International Commission on Radiological Protection (ICRP) recommended depth (*d*) = 10 mm. Based on this, in Japan, (*H**(10)) is called the 1 cm dose equivalent for work environments and (*H*_*p*_(10)) is the 1 cm dose equivalent for personnel; both are generally termed 1 cm dose equivalents.

#### 70 µm dose equivalent

For weakly penetrating radiation, the dose equivalent of a tissue 70 µm under the body surface (*d* = 0.07 mm) is used as the dose equivalent for the skin. The 70 µm dose equivalent for work environments and the 70 µm dose equivalent for personnel are generally termed 70 µm dose equivalents. Quantities for personnel are assessed using radiophotoluminescent glass dosimeters and other personal dosimeters.

#### Equivalent dose

*H*_T_ is the dose used to evaluate the effects of radiation on the tissues or organs, which is given by the formula below.

Where *D*_T,R_ is the mean absorbed dose in the tissue or organ (T) received from the radiation (*R*), and *W*_R_ is the radiation weighting factor of radiation (R). If there are multiple types of radiation, the sum of the values for each type will become the equivalent dose *H*_T_. The unit of equivalent dose is usually the sievert (Sv).

#### Dose limit

In the context of radioprotection, the dose limit is the effective dose or equivalent dose value that must not be exceeded in individuals. Dose limits in the existing laws and ordinances were established on the basis of ICRP recommendations (1990) [12]. The effective dose limit for personnel is 50 mSv per year and 100 mSv over 5 years. The value for the general public is 1 mSv per year. These values represent the total external and internal exposure, but do not include exposure to natural background radiation.

#### Dispersal rate

Factor used in calculating the concentration of radionuclides in discharged air or in the atmosphere.

Gas—using a gas trap 10^− 1^

Using an instrument other than a gas trap 1

Liquid, solid matter 10^− 3^

#### Occupancy factor

Ratio of the estimated dose received by a third party from a patient and the cumulative dose after an infinite time (the time until all nuclides have decayed) in a place 1 m away from the point source (patient) for the nuclide of interest.

#### Medical radioactive contaminated objects

Defined as matter contaminated by medical radionuclides, or radionuclides for positron emission tomography by the Ordinance for Enforcement of the Medical Care Act.

### Secular equilibrium for radionuclides

If a parent nuclide’s physical half-life is substantially longer than its progeny nuclide, the following relationships hold as a secular equilibrium:

1 $${\lambda _{\text{1}}}{N_{\text{1}}}={\lambda _{\text{2}}}{N_{\text{2}}}$$

2 $${A_{\text{1}}}={A_{\text{2}}}$$

3 $${N_{\text{1}}}/{T_{\text{1}}}={N_{\text{2}}}/{T_{\text{2}}}$$

Where

λ_1_: Decay constant of parent nuclide = 0.693/*T*_1_

λ_2_: Decay constant of progeny nuclide = 0.693/*T*_2_

*N*_1_ or *N*_2_: Number of atoms of parent nuclide or progeny nuclide at radiation equilibrium

*A*_1_ or *A*_2_: Radioactivity of parent nuclide or progeny nuclide at time *t*

*T*_1_ or *T*_2_: Physical half-life of parent nuclide or progeny nuclide

The number of atoms of Rn-219 at the secular equilibrium of 1 MBq Ra-223 can be obtained by applying equations (4) as modifications of (1)–(3).

4 $$A{\text{1}}={\text{ }}\lambda {\text{2}}N{\text{2}},N{\text{2}}=A{\text{1}}/\lambda {\text{2}},N{\text{2}}=A{\text{1}} \times T{\text{2}}/0.{\text{693}}$$

*A*1 = λ2*N*2, *N*2 = *A*1/λ2, *N*2 = *A*1 × *T*2/ 0.693

*N*_2_ = 1 × 10^6^ × (3.96/0.693) : 5.71 × 10^6^ [pieces]

*M =* 5.71 × 10^6^ [pieces] /6.02 × 10^23^ [pieces] /×22.4 × 1000 [mL] : 2.13 × 10^− 13^ [mL]

## Footnotes

1 11 MBq was calculated from the maximum dosage for one injection in the first clinical trial in Japan (110 kBq/kg x 100 kg).

2 The 11 MBq was calculated from the maximum dose for one injection in the first clinical trial in Japan (110 kBq/kg x 100 kg).
